# Mechanisms and therapeutic strategies of immune checkpoint molecules and regulators in type 1 diabetes

**DOI:** 10.3389/fendo.2022.1090842

**Published:** 2023-01-10

**Authors:** Jia-Tong Ding, Kang-Ping Yang, Kong-Lan Lin, Yu-Ke Cao, Fang Zou

**Affiliations:** ^1^ Department of Endocrinology and Metabolism, The Second Affiliated Hospital of Nanchang University, Nanchang, China; ^2^ The Second Clinical Medicine School, Nanchang University, Nanchang, China; ^3^ School of Ophthalmology & Optometry, Nanchang University, Nanchang, China

**Keywords:** immune checkpoints, immune checkpoint inhibitors, type 1 diabetes, lymphocyte, immunotherapy

## Abstract

**Background:**

Considered a significant risk to health and survival, type 1 diabetes (T1D) is a heterogeneous autoimmune disease characterized by hyperglycemia caused by an absolute deficiency of insulin, which is mainly due to the immune-mediated destruction of pancreatic beta cells.

**Scope of review:**

In recent years, the role of immune checkpoints in the treatment of cancer has been increasingly recognized, but unfortunately, little attention has been paid to the significant role they play both in the development of secondary diabetes with immune checkpoint inhibitors and the treatment of T1D, such as cytotoxic T-lymphocyte antigen 4(CTLA-4), programmed cell death protein-1(PD-1), lymphocyte activation gene-3(LAG-3), programmed death ligand-1(PD-L1), and T-cell immunoglobulin mucin protein-3(TIM-3). Here, this review summarizes recent research on the role and mechanisms of diverse immune checkpoint molecules in mediating the development of T1D and their potential and theoretical basis for the prevention and treatment of diabetes.

**Major conclusions:**

Immune checkpoint inhibitors related diabetes, similar to T1D, are severe endocrine toxicity induced with immune checkpoint inhibitors. Interestingly, numerous treatment measures show excellent efficacy for T1D *via* regulating diverse immune checkpoint molecules, including co-inhibitory and co-stimulatory molecules. Thus, targeting immune checkpoint molecules may exhibit potential for T1D treatment and improve clinical outcomes.

## 1 Introduction

Type 1 diabetes mellitus (T1D), which is regarded as an autoimmune disorder driven by T cells, causes a lack of insulin and exogenous insulin dependency as a result of the destruction of the patient’s islet cells by T cells (1–3). Despite exogenous insulin therapy representing an effective therapeutic strategy, the high morbidity and mortality of T1D cannot be ignored (4). Residual islet cells, which have received little attention, are still able to achieve glycemic control and reduce chronic inflammation, so immunomodulatory therapies targeting islet cells may be crucial for maintaining residual islet cells as the focus switches from exogenous insulin to endogenous insulin (5, 6). At the same time, the phase of remission (also known as honeymoon, partial remission, or PR) is increasingly being described as a phase of glycemic control and temporary recovery of islet β-cells that may occur after approximately 3 months of insulin therapy in T1D (7). PR may last 6-9 months, with a probability of occurrence of 35-43%, and this phase is likely to have a profound impact on the prognosis of T1D (8). Although the exact timing and mechanism of PR are not yet clear, there is still much research on how to personalize immunotherapy at this stage, such as the latest FDA approval of teplizumab, which is the first immunomodulator shown to significantly delay disease progression in high-risk individuals before a clinical episode (9–11).

It has been suggested that the absence of co-suppressive immune checkpoint ligands (e.g. PD-L1, HLA-E, CD86, and Gal-9) in β-cells in PR can significantly affect the development of T1D (12–14). Immune checkpoints (ICPs) are a series of molecules expressed on the surface of Treg cells and other immune cells that prevent the body from over-activating to the detriment of its normal cells (15). In early studies, immune checkpoint inhibitors (ICIs) (e.g. anti-CTLA-4, anti-PD-1, anti-PD-L1) were investigated in combination with ICPs, and ICPs could be used in the treatment of tumor immune escape through immune checkpoint blockade(ICB) therapy (15, 16). For T1D, activating the expression of ICPs to protect pancreatic islet β-cells from T-cell attack may have the potential to reverse early-onset T1D or to improve prognosis (9, 17, 18). ICPs can also protect human islet-like organ transplants from T-cell attack, induce antigen-specific immune tolerance and reverse early-onset hyperglycemia in bioengineered β-cells (6, 18). Notwithstanding, due to their endocrine toxicity, improper use of ICIs may raise the chance of developing T1D, and ICIs-induced diabetes mellitus, or ICI-associated diabetes, appears to be distinct from T1D, although this has to be proven by additional research (19–23). Interestingly, there is also evidence suggesting that T1D can affect the therapeutic effect of ICIs on tumors by altering the activity of ICPs (24).

The individual immunological checkpoints and co-regulators linked to autoimmune diabetes will be reviewed and discussed in detail in this study. Future research may be able to pinpoint ways to avoid T1D by examining the processes and pathways of ICPs to delay its onset or lessen its severity (25, 26). This article will also provide a summary of some of the benefits of using ICPs to treat secondary T1D as well as some possible clinical uses for ICPs.

## 2 Tregs cells in T1D

**Figure 1:**
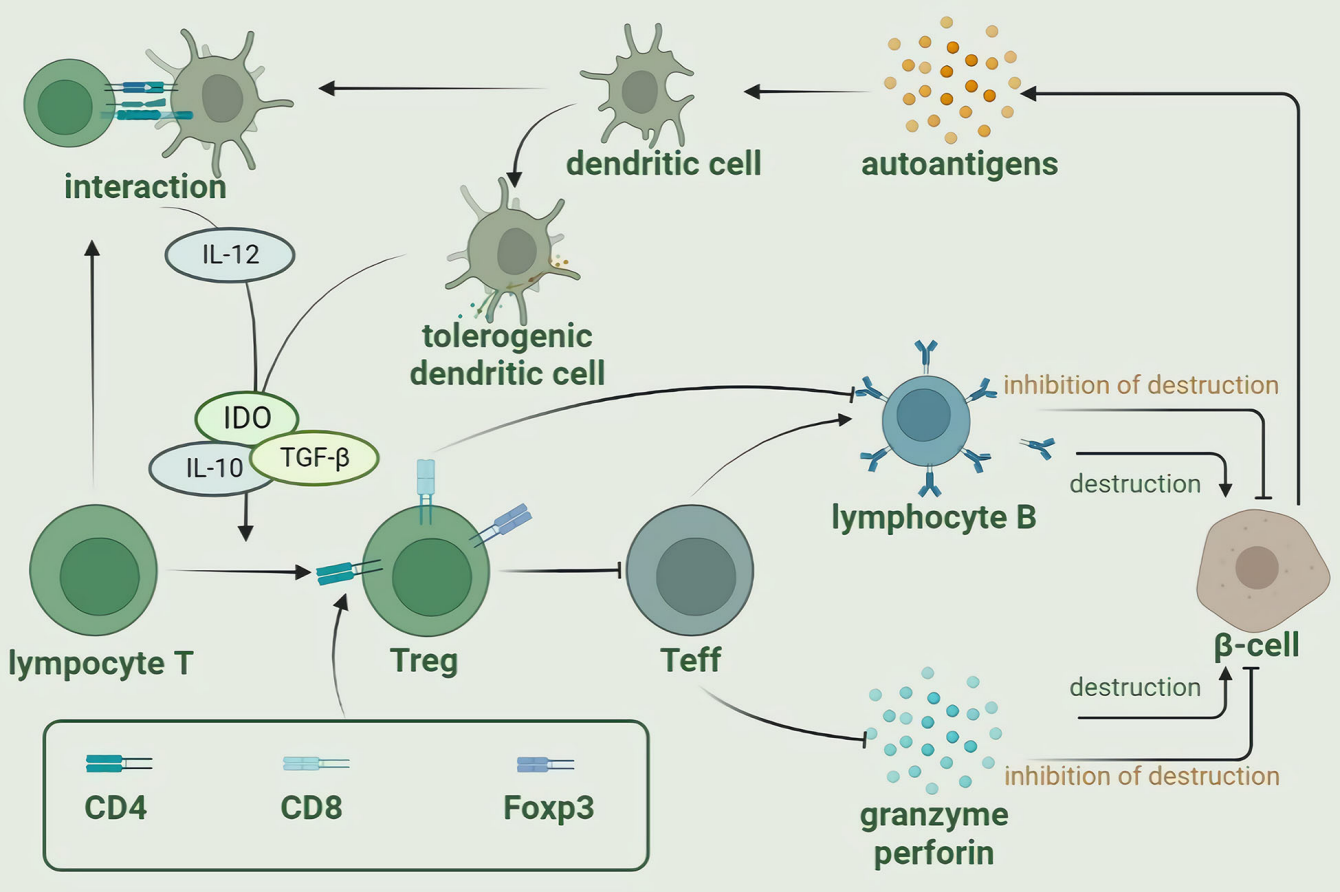
The regulatory roles of T cells in autoimmune reaction (created with Biorender).

Regulatory T cells (Tregs), also known as Foxp3+Tregs, are a class of suppressor T cells associated with the induction and maintenance of immune tolerance (27). When measured in peripheral blood using CD25 (the alpha chain of the IL-2 receptor) as a Tregs marker, a reduced number of T1D was found compared to the normal group (28). However, there was no change in Treg number by FOXP3 expression-defined Tregs peripheral blood frequency, and observation of the phenotype of Treg intercompartment in T1D patients also revealed no significant size change, pointing to the hypothesis that the major alteration of Treg in T1D is not numerical but rather its function (29, 30). Loss of Treg function has been attributed to pathways known to be critical for optimal Treg inhibitory function pathways, including the IL-2 and T cell receptor (TCR) pathways. A recent clinical study attempting to treat T1D using low-dose IL-2 in combination with Treg found a significant effect on Treg function maintenance while also neglecting the amplification of NK or CD8 T cells, which may be ameliorated by modification of IL-2 in the future (31). Tregs can be produced by three pathways: thymus-derived nTregs, peripheral *in vivo*-induced pTregs, and *in vitro*-induced Tregs, and inhibit the immune response function of APCs mainly through cellular contact, where receptors such as PD-1 or CTLA-4 on their surface will competitively bind ligands such as PD-L1/PD-L2, CD80/CD86 on antigen-presenting cells (APCs). Reduced function of Tregs leads to the development of T1D, with IL-10-induced chronic systemic hypo-inflammation state and Teff-mediated immune attack on β-cells, which will lead to the development of T1D (32, 33). Clinical trials using Treg have shown improved but not as promising results as expected, with only a few clinical studies showing that higher levels of Tregs and IL2 appear to improve endogenous insulin secretion in T1D, and the exploration of insulinogenic-specific Tregs in the immune response of patients with T1DM needs to continue in-depth (34–37). Therefore, how to use Tregs to target the autoimmunity against islet β cells that occurs in T1D has become a hot topic in scientific research (38, 39). In many studies using a mouse model of autoimmune diabetes, the use of IL-2 to modulate Treg was found to reduce interferon production by pancreatic infiltrating T cells, increase beta-cell numbers, and mitigate other immune therapies that interfere with Treg homeostasis and prevent disease (40–42). As research progresses, the mechanisms involved will be uncovered and more precise therapeutic modalities that do not produce off-target effects will be proposed (**Figure 1**).

### 2.1 Treg-cell transplantation

In earlier studies, nTregs were found to promote beta-cell regeneration through autologous transplantation, and patients could reduce the amount of exogenous insulin needed to maintain normal blood glucose levels (43). These studies point to the protective effect of Tregs on the islets, and the prolonged honeymoon period and reduced insulin dosage exhibited by patients confirm the effectiveness of Tregs (44, 45). Mechanistically, defects in Tr1 cells would lead to autoimmune diseases, that IL-10 prevents islet destruction and clinical symptoms of T1D through the production of cytokine pathways such as IFN-γ or IL-17, and early intervention of IL2, which can aid in the induction or maintenance of Foxp3, helps to re-establish the proper immune environment and slow down or even reverse the pathological process of T1D (33).

### 2.2 Induction of Tregs cells

In recent years, research has increasingly focused on increasing Tregs levels through stimulation of other cell subpopulations, cytokine interactions, or pharmacological treatments (46, 47). One way to increase Tregs is by stimulating other cell subsets using tolerogenic dendritic cells (DCs), which have an anti-inflammatory phenotype and can be induced by infusion of pathogenic DCs, or by using DCs to induce Tregs to proliferate *in vivo* and *in vitro (* 48, 49). It has been demonstrated that the relay transfer of DCs exposed to GM-CSF to naive mice leads to a significant delay in Foxp3+ T cell expansion and T1D onset. GM-CSF acts mainly on DCs and leads to the expansion of Foxp3+ Tregs, thus delaying the onset of T1D in NOD mice, and this inhibition may be mediated by enhanced IL-10 and TGF-β1 production (50). As for drugs, liru1 dexamethasone, vitamin D3 and rapamycin; or exposure to CTLA-4 membrane receptors and oligonucleotides exerted a slowing of the decline in β-cell function and improved HbA1c in recent-onset T1D (46, 48, 51).

### 2.3 Islet transplantation

Islet transplantation is an experimental treatment for T1D. As an experimental procedure, islet transplantation can only be performed as part of a clinical trial permitted by the U.S. Food and Drug Administration (FDA). Patients undergoing transplantation are often required to take long-term immunosuppressive drugs, which can be extremely harmful, and implantation-related foreign body reactions (FBR) often induce necrosis of the transplanted islets and lead to failure of glycemic control. The use of valproic acid (VPA) in short-chain fatty acids was shown to successfully protect islet grafts, prolong islet graft survival after islet transplantation, and increase IL-4-producing CD4 and Treg cell populations in NOD receptors, and VPA-induced Treg differentiation from juvenile CD4 and Treg cells by increasing the expression of transcription factor STAT5 and histone 3(H3) acetylation. CD4 and Treg cells. However, the hepatotoxicity, hyperammonemia, weight gain, and insulin resistance side effects of VPA cannot be ignored. To avoid this, the authors observed the same effectiveness of *in situ* transplantation using *in vitro* VPA-induced regulatory T cells, which also prolonged islet transplantation survival (52). However, a recent study has attempted to prepare a series of amphoteric-coated core-shell microcapsules (including carboxy betaine methacrylate [CBMA]-coated gelatin methacrylate [GelMA] [CBMA-GelMA], sulfobetaine methacrylate [SBMA]-coated gelMA [SBMA-GelMA] and methacrylic acid phosphorylcholine [MPC]-coated gelMA [MPC-GelMA]) and demonstrated their effectiveness in preventing protein adsorption, cell adhesion and inflammation *in vitro* (53).

## 3 ICP, ICI, and T1D

### 3.1 ICP and ICI in T1D

With the rise and wide application of immunotherapy in the field of tumor treatment, ICP and ICI have become research hotspots. ICP, immunosuppressive small molecules on the surface of T lymphocytes, prevent T cells from being overactivated by inhibiting T cell activation and downregulating immune responses, thereby protecting normal tissues from accidental injury, which is equivalent to installing a brake function on T cells (54, 55). With their flexible regulation of the duration and magnitude of physiological immune responses, ICPs play an indelible role in maintaining autoimmune homeostasis and immune tolerance (22). Common immune checkpoints include cytotoxic T lymphocyte-associated protein-4 (CTLA-4), programmed death-1 (PD-1) and T cell immunoglobulin and mucin domain-containing protein 3 (Tim-3) (56, 57). ICPs negatively modulate the immune response by binding to their ligands, such as PD-1 and PD-L1, but also thereby increase the potential for tumor immune evasion (58). Normally expressed on chronically activated T cells in peripheral tissues, PD-1 is also expressed on pancreatic islet cells. PD-1 transmits negative signals to T cells by binding to the ligand PD-L1 or PD-L2, thereby promoting the suppression of immune responses (59–63). Blocking the PD-1/PD-L1 pathway accelerated the risk of diabetes in non-obese diabetic (NOD) mice. But conversely, increasing the expression of PD-1/PD-L1 or using drugs to restore the PD-1/PD-L1 pathway reversed the course of diabetes in mice (64–66). In normal populations, the surface of islet cells expresses PD-1 for self-protection, and the surface of T cells also helps islet B cells expressing PD-1 bypass the immune response through ICP (62, 67). Furthermore, interferon is also a major regulator of PDL1 expression in human pancreatic β cells (68). In early pancreatitis, depending on the regulation of signal transducer and activator of transcription 1 (STAT1) and STAT2 genes, IFN-α can greatly increase the expression level of PD-L1 in pancreatic islet B cells (69, 70). During islet inflammation, PDL1 expression in β-cells is upregulated by a mechanism thought to be induced by type I and type II IFNs (12, 68). This suggests that pancreatic islet β-cells try to downregulate the immune response by upregulating PDL1, thus avoiding further tissue damage. However, if the PD-1/PDL1 pathway is blocked, it will break immune tolerance, and, ultimately, lead to the development of T1D (71, 72). This was confirmed in animal experiments in NOD mice: higher levels of PDL1 were detected in B cells that survived the immune attack, and high levels of PDL1 were also found to reduce the incidence of diabetes in NOD mice (73, 74).

Immune checkpoint inhibitors work by inhibiting the “off-duty” signal from tumor cells, restoring the immune system to function normally, and then attacking the tumor cells. ICIs block the binding of the ICP to its ligand, to overcome the inhibitory effect, unleash the suppressive function, and reactivates the specific immune function of T lymphocytes against cancer (75, 76). After the binding of PD-1 expressed on the surface of T cells to the ligand is inhibited, those self-reactive T cells that target islet cells are activated to attack their islet cells, and the islet cells are destroyed, resulting in decreased insulin secretion (63). Anti-PD-1 drugs induced PD-1 reduction may also activate autoimmune T cells, leading to autoimmune inflammation targeting pancreatic islet cells (67, 77, 78). In summary, with PD-1/PD-L1 inhibitors, the expression level of PD-L1 on B cells will be greatly reduced, which makes B cells lose their armor against autoimmune attacks. With continued loss of cells, T1D will be the inevitable result. And such events may be more frequent in individuals with susceptibility genotype, HLA haplotypes for example (62, 79).

### 3.2 Immune checkpoint inhibitor-induced type 1 diabetes (ICIT1D)

When the suppressive effects of T cell immunity are removed, T cells become hyperactivated, and the body mounts an autoimmune response, leading to a unique set of immune-related adverse events (irAEs) (80–83). IAE often affects the endocrine system and results in a variety of endocrine disorders, including hypophysitis, thyroid dysfunction, insulin deficiency diabetes, PAI, etc (21, 84). Relatively speaking, immune checkpoint inhibitor-induced type 1 diabetes (ICIT1D) is a comparatively rare type of adverse reaction with the incidence of ICIT1D equaling 1% (54, 85). However, ICIT1D induces life-threatening diabetic ketoacidosis (DKA) without timely treatment in about 38% to 71% of patients and may serve as a high-risk factor for adrenal crisis (86, 87).

**Figure 2:**
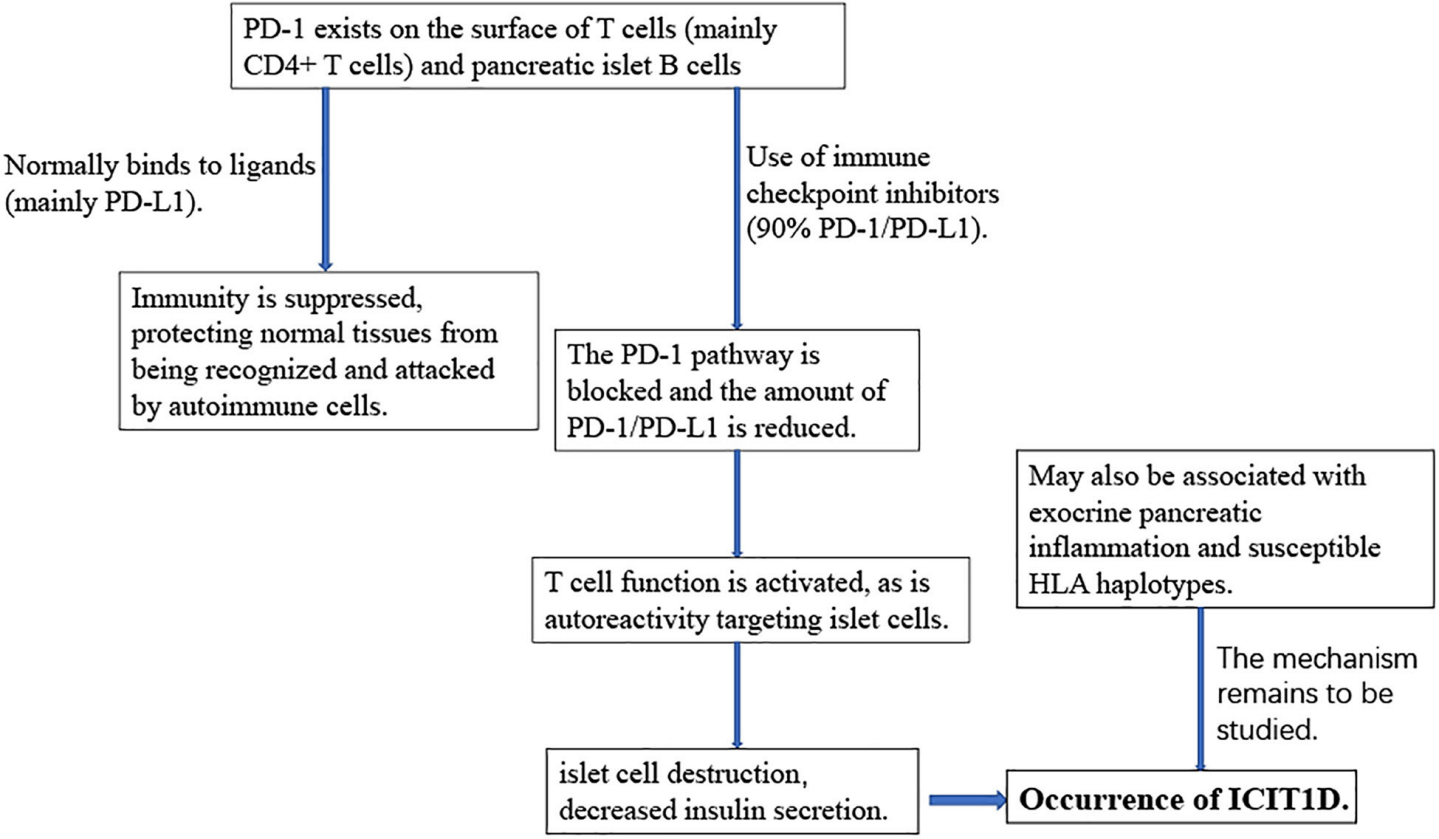
The roles of the PD-1/PD-L1 pathway in ICI-induced T1D.

Understanding the pathogenesis of ICIT1D plays an important role in its prevention and treatment (**Figure 2**). ICIT1D can be divided into four distinct entities: acute autoimmune insulin−dependent diabetes, the clinical presentation of type 1 diabetes, the complication of autoimmune pancreatitis, and autoimmune lipoatrophy, the first of which is most frequently reported (60, 88). ICIT1D usually manifests as a rapid, sustained, severe drop in insulin, C-peptide, and blood glucose levels, with a higher age of onset, faster progression, and less antibody positive compared with traditional T1D (25, 86, 89–91). Immunological characteristics indicate that patients with ICIT1D have humoral and cellular autoimmunity, and some patients may have islet autoantibodies, providing evidence for the involvement of autoimmune mechanisms (26, 62, 92). Based on a colossal number of case studies, more than three-quarters of all ICIT1D cases are associated with treatment with PD-1 inhibitors. Combined use of PD-1 and CTLA-4 inhibitors accounted for 17%, PD-L1 inhibitors for 6%, and very few were associated with CTLA-4 monotherapy (3%) (25, 63, 89, 91, 93).

## 4 Potential clinical application of immune checkpoint molecules in T1D

**Figure 3:**
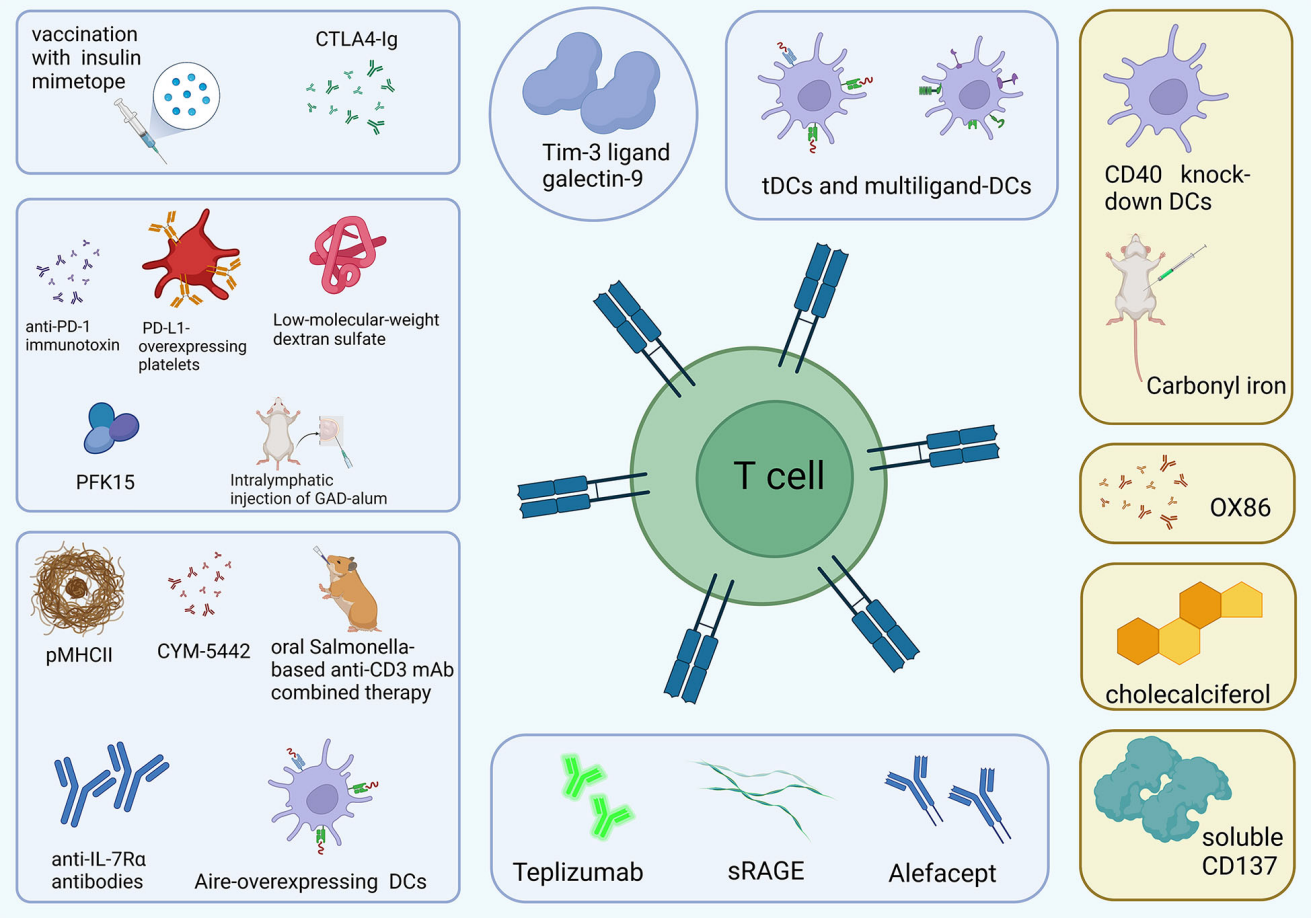
The treatment measures for T1D *via* regulating T cells. The blue rectangles represent co-inhibitory molecules while the yellow rectangles represent co-stimulatory molecules (created with Biorender).

Although T1D is considered one of the annoying side effects induced by immune checkpoint inhibitors, immune checkpoint molecules exhibit the potential as a therapeutic maneuver for T1D control (20, 89, 94). In general, inhibition of autoreactive lymphocyte populations is considered an effective therapeutic strategy for the treatment of autoimmune diseases including T1D, but its clinical application is limited due to the massive suppression of lymphocytes involved in normal adaptive immunity (17, 95, 96). Therefore, targeted inhibition of pathogenic lymphocytes associated with autoimmune diseases generated considerable clinical interest (97, 98). Unlike most existing immune suppressants, inhibition of T-cell stimulation *via* blocking specific signaling molecules focuses on activated lymphocytes and preserves normal adaptive immunity (97). Immune checkpoint molecules perform as a strong immune regulator of self-tolerance and autoimmunity and regulate the response of various immune cells, including T cells, natural killer cells, dendritic cells, innate lymphoid cells, macrophages, and myeloid cells, and specific immune checkpoint mechanisms also participate in pathological processes of T1D (99–101). A recent cohort study also showed that higher levels of circulating immune checkpoint molecules, especially CD137/4-1BB and PD-1, may serve as prognostic biomarkers for new-onsets T1D and risk factors for the growth of an additional autoimmune disease (102). Considering the above characteristics, what follows in the passage reviews the possible therapeutic implications in T1D *via* regulating immune checkpoint molecules (**Figure 3**), including co-inhibitory molecules (**Table 1**) and co-stimulatory molecules (**Table 2**), especially CTLA-4, PD-1, LAG3, and TIGIT, which seems to be considered as pivotal regulatory molecules with excellent clinical application value.

**Table 1:** The roles of treatment measures regulating co-inhibitory molecules for T1D.

Treatment measures	Related mechanisms	Ref.
CTLA-4		
CTLA4-Ig	Selectively block CD28 co-stimulation and defend against potentially autoreactive T cells.	(7, 103)
sub-immunogenic vaccination with strong agonistic insulin mimetope	Suppress effector T cells *via* inducing human insulin-specific Foxp3+ Treg with upregulated Foxp3, CTLA4, IL-2Rα, and TIGIT expression.	(104)
PD-1		
anti-PD-1 immunotoxin	Selectively target and deplete PD-1-expressing cells.	(97)
Genetically engineered PD-L1-overexpressing platelets	Suppress autoreactive pancreatic T-cell activity and reverse diabetes in novel hyperglycemic NOD mice.	(17)
PFK15	Increase the expression of PD-1 and LAG-3 on CD4 T cells, inhibit glycolysis utilization of diabetogenic CD4 T cells and reduce T cell responses to β cell antigen.	(105, 106)
Low-molecular-weight dextran sulfate	Increase PD-1 expression in T cells, reduce interferon-γCD4 and CD8 T cells, and enhance the number of foxp3 cells.	(107, 108)
Intralymphatic injection of aluminum-formulated glutamic acid decarboxylase (GAD-alum)	Delay the progression of T1D with immunomodulatory effects including increased PD-1+ CD69+ cells in both CD8+ and double negative T cells.	(109, 110)
LAG3		
stable peptide-MHC class II complexes (pMHCII)	Induce T cell suppression and thereby inhibit diabetes progression in NOD mice with LAG-3 deficiency.	(111)
CYM-5442	Induce the expression of negative immune regulator receptor genes Pdcd1, Lag3, Ctla4, Tigit, and Btla to inhibit the autoimmune ability of T cells	(112)
oral Salmonella-based anti-CD3 mAb combined therapy	Immunosuppressive CD4CD25Foxp3 Treg and CD4CD49bLAG3 Tr1 cells.	(113)
anti-IL-7Rα antibodies	Delay T1D incidence and upregulates LAG-3, Tim-3, and PD-1 on peripheral blood CD4 and CD8 T cells from prediabetic NOD mice.	(114)
Aire-overexpressing DCs	Upregulate CD73, Lag3, and FR4 that mediate self-tolerance, and decrease CD4^+^IFN-γ^+^ autoreactive T cells in STZ-T1D mouse-derived splenocytes, which is associated with Aire-overexpressing DCs-induced T1D prevention and delay.	(115)
Tim		
Tim-3 ligand galectin-9	Enhance apoptosis of CD4+ Tim-3+ Th1 cells and downregulate Th1 immune response, and anti-Tim-3 monoclonal RMT3-23 antibody suppresses the TNF-α production and activation of DC.	(116, 117)
TIGIT		
Teplizumab	Increase the percentage of KLRG1^+^TIGIT^+^CD8^+^ T cells.	(118)
soluble receptor for advanced glycation end products (sRAGE)	Reverse RAGE ligand-induced downregulation of key genes for Treg homeostasis and activation, including FOXP3, IL7R, TIGIT, JAK1, STAT3, STAT5b, and CCR4.	(119, 120)
Alefacept	Hypo proliferative CD8 memory cells expressing exhaustion-associated markers including TIGIT and KLRG1.	(121)
BTLA-HVEM		
tDCs and multiligand-DCs	Suppress the function of effector T cells and induce self-tolerance.	(122)

**Table 2:** The roles of treatment measures regulating co-stimulatory molecules for T1D.

Treatment measures	Related mechanisms	Ref.
CD40		
CD40 knock-down DCs	Improve blood glucose, glucose tolerance, weight, and IL-13 production.	(123)
Carbonyl iron	Decreases the expression of CD40 and CD80 on DCs to suppress antigen-presenting ability and further adaptive immune response toward pancreatic beta cells.	(124)
OX40		
OX40 agonistic antibody (OX86)	Induce CD4+CD25+Foxp3+ Tregs and CD4+Foxp3- T cells expressing the latency-associated peptide play a synergistic role with insulin B9:23.	(125)
ICOS		
cholecalciferol supplement	Increase 25(OH)D levels and decreases Th17 and Treg/ICOS+ percentages in the serum of healthy siblings.	(126)
4-1BB		
soluble CD137	Induce CD4+ T cell anergy, in turn suppressing antigen-specific T cell proliferation and IL-2/IFN-γ production.	(127)

### 4.1 Co-inhibitory molecules

CTLA-4 belongs to the immunoglobulin-related receptor family that is involved in multiple aspects of T cell immune regulation and peripheral tolerance and is considered to be one of the non-HLA genetic markers for T1D susceptibility (128–131). Due to a homolog structure to CD28, CTLA4 proteins share common ligands with CD28(CD80 and CD86) and even have higher affinity to CD80 and CD86 (132, 133). Therefore, CTLA4 proteins negatively regulate CD28-mediated T-cell co-stimulation (133, 134). Based on these characteristics, CTLA4-immunoglobulin (Ig), namely abatacept, selectively blocks CD28 co-stimulation and defends against potentially autoreactive T cells, thereby preventing T1D (7, 103, 132). Serr et al. have reported that sub-immunogenic vaccination with strong agonistic insulin mimetope efficiently suppresses effector T cells *via* inducing human insulin-specific Foxp3+ Treg with upregulated Foxp3, CTLA4, IL-2Rα and TIGIT expression, which provides a potential new drug target for prevention of islet autoimmunity of T1D (104).

PD-1(CD80), one member of the immunoglobulin superfamily, negatively regulates immune responses and mediates immune tolerance, impact on disease progression and aetiology of T1D (135–137). PD-1 deficiency specifically accelerates the development of subacute T1D in NOD mice (136, 138, 139). Downregulation of PD-1/PD-L1 on CD4+ and CD8+ T cells in patients with T1D is dynamically recovered in partial remission but decreased again after the partial remission phase (140, 141). Thus, the above-mentioned results suggest that PD-1/PD-L1 may be a potential target for T1D therapy. An anti-PD-1 immunotoxin selectively targeting and depleting PD-1-expressing cells delays disease onset in mouse models of autoimmune diabetes (97). Genetically engineered PD-L1-overexpressing platelets also suppress autoreactive pancreatic T-cell activity and reverse diabetes in novel hyperglycemic NOD mice (17). PFK15 treatment has been reported to increase the expression of PD-1 and LAG-3(lymphocyte-activation gene 3) on CD4 T cells and prevent the development of diabetes *via* inhibiting glycolysis utilization of diabetogenic CD4 T cells and reducing T cell responses to β cell antigen (105, 106). Low-molecular-weight dextran sulfate reduces the incidence of diabetes and even reverses diabetes in early-onset diabetic NOD mice, at least partly *via* increasing PD-1 expression in T cells, reducing interferon-γCD4 and CD8 T cells, and enhancing the number of FoxP3 cells (107, 108). Intralymphatic injection of aluminum-formulated glutamic acid decarboxylase (GAD-alum) delays the progression of T1D with immunomodulatory effects including increased PD-1+ CD69+ cells in both CD8+ and double negative T cells (109, 110).

Apart from CTLA-4 and PD-1, the next wave of co-inhibitory immune checkpoint receptor targets, including Lag-3, Tim-3, and TIGIT, are drawing increasing attention in clinical application.

LAG3 is an inhibitory immune checkpoint receptor regulating multiple immune functions, including T cell activation and proliferation, cytokine production, and cytolytic activity (142, 143). LAG-3 blockade promotes disease growth and progression in autoimmune-prone models. Corresponding to this, Jones et al. reported that T1D patients exhibited fewer LAG-3 CD4 and CD8 T cells compared with healthy controls (144). The interaction between LAG-3 and stable peptide-MHC class II complexes (pMHCII) induces T cell suppression and thereby inhibits diabetes progression in NOD mice with LAG-3 deficiency (111). Moreover, CYM-5442, a selective S1PR1 agonist, induces the expression of negative immune regulator receptor genes Pdcd1, Lag3, Ctla4, Tigit, and Btla to inhibit the autoimmune ability of T cells, leading to T1D prevention in the mouse Rip-LCMV T1D models (112). Similarly, oral Salmonella-based anti-CD3 mAb combined therapy tempts immunosuppressive CD4CD25Foxp3 Treg and CD4CD49bLAG3 Tr1 cells, then contributing to reversion of new-onset T1D in NOD mice (113). Treatment with anti-IL-7Rα antibodies for two weeks delays T1D incidence and upregulates LAG-3, Tim-3, and PD-1 on peripheral blood CD4 and CD8 T cells from prediabetic NOD mice (114). Autoimmune regulator (Aire)-overexpressing DCs upregulates CD73, Lag3, and FR4 that mediate self-tolerance, and decreases CD4^+^IFN-γ^+^ autoreactive T cells in STZ-T1D mouse-derived splenocytes, which is associated with Aire-overexpressing DCs induced T1D prevention and delay (115).

Tim-1, Tim-3, and Tim-4 are members of the T-cell immunoglobulin and mucin domain (Tim) molecule family in humans, and mediate peripheral immune tolerance *via* interacting with its ligands (145, 146). Compared with healthy controls, upregulated Tim-1 and downregulated Tim-3 led to imbalanced ratios of Tim-3/Tim-1 in T1D, in particular T1D patients with defective islet function (147). Another research focusing on Tregs reveals that Tim1 and Tim4 on CD4CD25 T cells decreased in peripheral blood mononuclear cells of patients with T1D than healthy volunteers (148). Tim-3 ligand galectin-9 enhances apoptosis of CD4+ Tim-3+ Th1 cells and downregulates Th1 immune response, and anti-Tim-3 monoclonal RMT3-23 antibody suppresses the TNF-α production and activation of DC, both exhibiting significant therapeutic effects on T1D (116, 117). Although a few studies addressing the therapeutic influence of Tim-related pathways for T1D in the last decade, we considered Tim as a novel target worthy of further exploration for treatment.

Expressed on Treg cells, T cell immunoglobulin and ITIM domain (TIGIT) is an inhibitory receptor that participates in the pathogenesis of T1D (149, 150). Higher percentage and expression levels of TIGIT are identified on CD4+CD25hi T cells, CD4+CD25- T cells, total CD4+ T cells, and non-CD4+ cells of peripheral blood mononuclear cells from T1D patients versus healthy controls (151). Teplizumab treatment increases the percentage of KLRG1+TIGIT+CD8+ T cells and suppresses disease progression to T1D in high-risk participants (118). A low circulating level of soluble receptors for advanced glycation end products(sRAGE) is representative of the high risk of T1D. sRAGE treatment reverses RAGE ligand-induced downregulation of key genes for Treg homeostasis and activation, including FOXP3, IL7R, TIGIT, JAK1, STAT3, STAT5b, and CCR4 (119, 120). Alefacept preserves endogenous insulin C-peptide production of T1D patients to a certain extent, which is related to hypo proliferative CD8 memory cells expressing exhaustion-associated markers including TIGIT and KLRG1 (121).

Another inhibitory immune checkpoint, BTLA (B- and T-lymphocyte attenuator)-HVEM (Herpesvirus entry mediator, namely TNFRSF14) complex, has been drawing increasing attention of the academic community as an important regulator in autoimmune reactions (152, 153). A lower expression of BTLA is identified in the peripheral blood of patients with young-onset T1D compared with adult-onset T1D patients (154). To explore a new therapeutic strategy, Gudi et al. conduct engineered tolerogenic dendritic cells (tDCs) expressing CTLA4 selective ligand and multiligand-DCs expressing a combination of CTLA4, PD1, and BTLA selective ligands, both of which present pancreatic β-cell antigen (BcAg). Both two types of engineered DCs, multiligand-DCs in particular, suppress the function of effector T cells and induce self-tolerance, thereby delaying the progression of T1D (122).

### 4.2 Co-stimulatory molecules

CD40 is a member of the tumor necrosis factor (TNF) receptor superfamily and interacts with CD40L to mediate the interaction between B and CD4+ T cells for germinal center responses and B cell activation (155, 156). Fully functional CD40 expression is not only required for hyperglycemia and insulitis in T1D but also induces relatively broad T‐cell receptor repertoire on CD40+ CD4+ cells (Th40 cells) during diabetogenesis (157). The number of Th40 cells significantly expands in the peripheral blood of T1D patients. Furthermore, Th40 cell levels also stratify pre-diabetic patients into two groups, with Th40-high subjects showing a higher percentage of disordered glucose tolerance, CD4/CD8 double-positive population, and T1D-associated HLA, including HLA DR4/DR4 and DQ8/DQ8 (158, 159). In the streptozotocin-induced diabetic mice model, CD40 knock-down DCs treatment improves blood glucose, glucose tolerance, weight, and IL-13 production (123). Highly expressed in B cells, Toll-like receptor 9(TLR9) is related to matrix metalloproteinases, tissue inhibitors of metalloproteinase-1, and CD40. B-cell-specific deletion of TLR9 near-completely protect NOD mice from T1D development (160). Adjuvant carbonyl iron inhibits the development of diabetes and decreases the expression of CD40 and CD80 on DCs to suppress antigen-presenting ability and further adaptive immune response toward pancreatic beta cells (124).

OX40, also named CD134 or TNFRSF4, a member of the TNF receptor family, serves as a co-stimulatory factor during T cell activation and controls effector and memory T cell responses (161–163). In T1D patients, soluble OX40 and OX40L expression in the serum is significantly upregulated and considered as potential indicators for disease progression, while membrane OX40 and OX40L expression on immune cells is significantly downregulated compared with the healthy controls (164). OX40 agonistic antibody (OX86) treatment induces CD4+CD25+Foxp3+ Tregs and CD4+Foxp3- T cells expressing the latency-associated peptide and reduces T1D incidence of NOD mice, which play a synergistic role with insulin B9:23. Interestingly, Tregs gathered from NOD mice treated with OX86 and insulin B9:23 also prevent T1D development when adoptively transferred into recipient mice (125).

Inducible co-stimulator (ICOS), a member of the CD28 superfamily, is expressed on activated T cells and specifically binds with its unique ligand ICOSL (165, 166). Children with impaired glucose tolerance and T1D exhibit a higher frequency of CXCR5+PD-1+ICOS+, CD4+CXCR5+, and CD4+CXCR5+ICOS+ circulating follicular helper T cells (Tfh). Interestingly, the expansion of CXCR5+PD-1+ICOS+ Tfh is more apparent in children with two or more biochemical autoantibodies (167, 168). Progressively reduction and suppression of ICOS+Foxp3+ Treg cells in islets are representing exacerbated T1D. Consistently, inhibited ICOS pathway also correlates with T1D progression in NOD.BDC2.5 mice (169). A recent cohort study exploring the potential association between blood serum 25 OH vitamin D(25[OH]D) levels and Th17 and Treg, and Treg/ICOS+ levels in healthy siblings of children with T1D reveals that Treg/ICOS+ percentages are higher in siblings with lower 25(OH)D levels and higher genetic risk for T1D. Furthermore, cholecalciferol supplement for 6 months increases 25(OH)D levels and decreases Th17 and Treg/ICOS+ percentages in the serum of healthy siblings (126). ICOS expression may also impact the effects of co-stimulation blockade administration. For example, higher frequencies of ICOS+ Tfh at baseline predict a poor clinical outcome following abatacept treatment (170).

As a member of the TNF receptor superfamily, 4-1BB, namely CD137 and TNFRSF9, is expressed on activated T cells and interacts with CD137L, the ligand of CD137, expressed by antigen-presenting cells (171). Interestingly, the impacts of CD137 on T1D progression in NOD mice associate with where it is expressed. CD137 in CD4 T cells suppresses T1D development, while CD137 expressed in CD8 T cells promotes disease progression (172). NOD.Tnfsf9-/- strain shows delayed T1D progression, less insulitis, and reduced β-cell-autoreactive CD8 T cells frequencies (173). Itoh et al. have reported that soluble CD137 induces CD4+ T cell anergy, in turn suppressing antigen-specific T cell proliferation and IL-2/IFN-γ production, thereby delaying progression to end-stage T1D in NOD mice (127).

## 5 Conclusion

ICIs block immune checkpoints and have emerged as a valuable alternative treatment for cancers with advanced stage, but endocrine toxicity, ICI-related DM, for example, limits their potential clinical application to some extent (20, 174). However, the similarity between ICI-related DM and T1D also suggests the potential feasibility of targeting immune checkpoint molecules for T1D treatment, which is also supported by higher circulating immune checkpoint molecule levels in T1D patients. Differing from massive immune inhibitors, targeted regulation of immune checkpoint molecules may specifically inhibit pathogenic lymphocytes associated with T1D (17, 96). Due to numerous co-inhibitory and co-stimulatory molecules involved in the treatment of T1D as mentioned above, it was valuable to explore novel therapeutic approaches regulating autoimmune-related T lymphocytes based on these targets for the management and treatment of T1D and may improve clinical outcomes.

## Author contributions

All authors have discussed the proposed scope and content of the article before drafting. J-TD, K-PY, and K-LL wrote and revised the paper. Y-KC collected literature. FZ reviewed and edited the manuscript. All authors contributed to the article and approved the submitted version.
